# Chlorate Specifically Targets Oxidant-Starved, Antibiotic-Tolerant Populations of Pseudomonas aeruginosa Biofilms

**DOI:** 10.1128/mBio.01400-18

**Published:** 2018-09-25

**Authors:** Melanie A. Spero, Dianne K. Newman

**Affiliations:** a Division of Biology and Biological Engineering, California Institute of Technology, Pasadena, California, USA; b Division of Geological and Planetary Sciences, California Institute of Technology, Pasadena, California, USA; National Cancer Institute

**Keywords:** Nar, *Pseudomonas aeruginosa*, antibiotic tolerance, biofilms, chlorate, nitrate reduction, prodrug

## Abstract

Nitrate respiration is a widespread mode of anaerobic energy generation used by many bacterial pathogens, and the respiratory nitrate reductase, Nar, has long been known to reduce chlorate to the toxic oxidizing agent chlorite. Here, we demonstrate the antibacterial activity of chlorate against Pseudomonas aeruginosa, a representative pathogen that can inhabit hypoxic or anoxic host microenvironments during infection. Aerobically grown P. aeruginosa cells are tobramycin sensitive but chlorate tolerant. In the absence of oxygen or an alternative electron acceptor, cells are tobramycin tolerant but chlorate sensitive via Nar-dependent reduction. The fact that chlorite, the product of chlorate reduction, is not detected in culture supernatants suggests that it may react rapidly and be retained intracellularly. Tobramycin and chlorate target distinct populations within metabolically stratified aggregate biofilms; tobramycin kills cells on the oxic periphery, whereas chlorate kills hypoxic and anoxic cells in the interior. In a matrix populated by multiple aggregates, tobramycin-mediated death of surface aggregates enables deeper oxygen penetration into the matrix, benefiting select aggregate populations by increasing survival and removing chlorate sensitivity. Finally, *lasR* mutants, which commonly arise in P. aeruginosa infections and are known to withstand conventional antibiotic treatment, are hypersensitive to chlorate. A *lasR* mutant shows a propensity to respire nitrate and reduce chlorate more rapidly than the wild type does, consistent with its heightened chlorate sensitivity. These findings illustrate chlorate’s potential to selectively target oxidant-starved pathogens, including physiological states and genotypes of P. aeruginosa that represent antibiotic-tolerant populations during infections.

## Abstract (precis)

The anaerobic growth and survival of bacteria are often correlated with physiological tolerance to conventional antibiotics, motivating the development of novel strategies targeting pathogens in anoxic environments. A key challenge is to identify drug targets that are specific to this metabolic state. Chlorate is a nontoxic compound that can be reduced to toxic chlorite by a widespread enzyme of anaerobic metabolism. We tested the antibacterial properties of chlorate against Pseudomonas aeruginosa, a pathogen that can inhabit hypoxic or anoxic microenvironments, including those that arise in human infection. Chlorate and the antibiotic tobramycin kill distinct metabolic populations in P. aeruginosa biofilms, where chlorate targets anaerobic cells that tolerate tobramycin. Chlorate is particularly effective against P. aeruginosa *lasR* mutants, which are frequently isolated from human infections and more resistant to some antibiotics. This work suggests that chlorate may hold potential as an anaerobic prodrug.

## INTRODUCTION

Nitrate respiration is a widespread mode of anaerobic energy generation that bacterial pathogens use to adapt to anoxic host environments (1, 2). Not only is nitrate one of the most energetically favorable electron acceptors used in anaerobic respiration, it is available at various sites in the human body, deriving from reactions between nitric oxide and superoxide produced during the host inflammatory response (3). In the gut, enteric pathogens respire host-derived nitrate to outcompete the obligate anaerobes that typically dominate this environment, contributing to inflammatory bowel disease (4–6). In Mycobacterium tuberculosis and its relatives, there is evidence that the respiration of macrophage-derived nitrate is required for growth, virulence, and persistence (7–9). Pathogenic species of *Staphylococcus*, *Burkholderia*, and *Brucella* also employ nitrate respiration to adapt to host-like environments, although the *in vivo* role of this physiology is not fully understood (10–14). The prevalence of nitrate respiration in bacterial pathogenesis marks this mode of energy generation as a potential therapeutic target for disparate infections.

Included among pathogens known to respire nitrate is Pseudomonas aeruginosa, which causes a range of acute and chronic infections (15). P. aeruginosa infections contribute to chronic wounds, ventilator-associated pneumonia, and the morbidity and mortality of patients with cystic fibrosis (CF), in whom these infections persist despite aggressive antibiotic treatment (16–18). Nitrate respiration likely supports P. aeruginosa growth and survival in the host, because microenvironments within chronic wounds and the sputum in CF patient lungs contain appreciable anoxic zones and nitrate concentrations (19–23). Although P. aeruginosa has three nitrate reductases (Nar, Nap, and Nas) (24), only the Nar enzyme couples nitrate reduction to proton translocation and is required for anaerobic growth (25). Transcription of the *nar* operon is activated under hypoxic/anoxic conditions and further stimulated by the presence of nitrate (25), so it is unsurprising that *nar* gene expression (26, 27) and the metabolic products of nitrate respiration have been detected in CF patient sputum (21, 28) and that Nar antibodies have been detected in the sera of CF patients (29).

One reason that P. aeruginosa infections might persist for decades despite antibiotic use is that oxidant-starved pathogen populations can be physiologically tolerant of antibiotics. Low-oxygen tensions in host environments limit the growth rates of pathogens (21, 30, 31), and slowly growing P. aeruginosa cells show increased physiological tolerance toward some classes of antibiotics (32, 33). Diverse reasons can explain this tolerance: (i) conventional drug targets are inappropriate (e.g., cell division inhibitors harm only cells that are actively dividing and may not harm slowly growing cells) (34), (ii) common antibiotics cannot be taken up (e.g., aminoglycosides require active transport, but if the cell membrane is insufficiently charged to allow these transporters to work, they will not enter the cell [35–37]), and (iii) target pathogens are physically sequestered and thereby less accessible to the immune system or conventional drugs (38). Many pathogens, including P. aeruginosa, also grow as aggregate biofilms in host sputum and tissue or on medical implants or catheters (39, 40). Biofilms are physiologically heterogeneous; exterior cells with access to oxygen respire aerobically, whereas interior cells experience hypoxia or anoxia, display markers of slow growth, and are physiologically tolerant of antibiotics used to treat P. aeruginosa infections, such as the aminoglycoside tobramycin (41–43). P. aeruginosa cells require a threshold membrane potential for aminoglycoside uptake, and antibiotic-tolerant populations can be sensitized via the addition of an electron acceptor or a carbon source that stimulates metabolic activity (44, 45).

Genetic changes accumulate in P. aeruginosa over the course of infection, and strains with inactivating mutations in *lasR* are known to arise rapidly in some host environments (46, 47). *lasR* mutants have been isolated from patients with bacteremia, pneumonia, chronic wounds, and CF (48–50). The prominence of *lasR* mutants has been best documented in CF studies, where they are among the most frequently isolated mutants from CF patients (49) and their presence is associated with worse lung function (46). *lasR* mutants are also more resistant to antibiotics commonly used to treat P. aeruginosa infections (51, 52). *lasR* encodes a quorum-sensing regulator, so the loss of this gene has pleiotropic effects (52), but intriguingly, one phenotypic trait of *lasR* mutants is their decreased rates of oxygen respiration and increased rates of Nar-dependent nitrate respiration (51). Selection for mutants that, besides having other traits, are metabolically rewired toward nitrate respiration supports the hypothesis that this mode of anaerobic energy generation plays an important role for P. aeruginosa *in vivo*. Because oxidant-starved and genetic (e.g., *lasR* mutant) populations are more antibiotic tolerant and synthesize Nar, we hypothesized that targeting nitrate respiration might provide a new approach for treating P. aeruginosa infections.

Chlorate has long been known to serve as a substrate for Nar. Since the 1960s, Nar activity has been distinguished from other nitrate reductases, such as Nap, by its ability to also reduce chlorate to chlorite (53, 54). While chlorate is a relatively stable, nontoxic compound, chlorite is a toxic, reactive oxidizing agent (55, 56). Thus, chlorate is expected to be nontoxic to cells lacking Nar (e.g., mammalian cells) and specifically kill Nar-containing bacterial cells via cytoplasmic chlorite production. Consistent with this logic, chlorate has been successfully used to control pathogens in studies of agricultural systems, where livestock that ingested chlorate had lowered intestinal and fecal enteric-pathogen counts (57). Importantly, the treated animals showed no measurable health effects with very high quantities of ingested chlorate (e.g., 250 mg kg^−1^ of body weight per day [58]), including in one study in which serum chlorate concentrations reached 1 mM in sheep (59). Reports also indicate that chlorate shows low toxicity in humans, with an estimated lethal oral dose of 20 to 35 g (60, 61).

Considering the central role that Nar-mediated nitrate respiration likely plays in the survival of bacterial pathogens in diverse host environments, we reasoned that chlorate treatment might offer an effective means of targeting anaerobic populations, such as those within the oxidant-limited interiors of biofilms. Here, we begin to test this hypothesis by exploring the antibacterial activity of chlorate against different physiological states of P. aeruginosa.

## RESULTS

### Chlorate kills *P. aeruginosa* cultures displaying physiological tobramycin tolerance.

**FIG 1:**
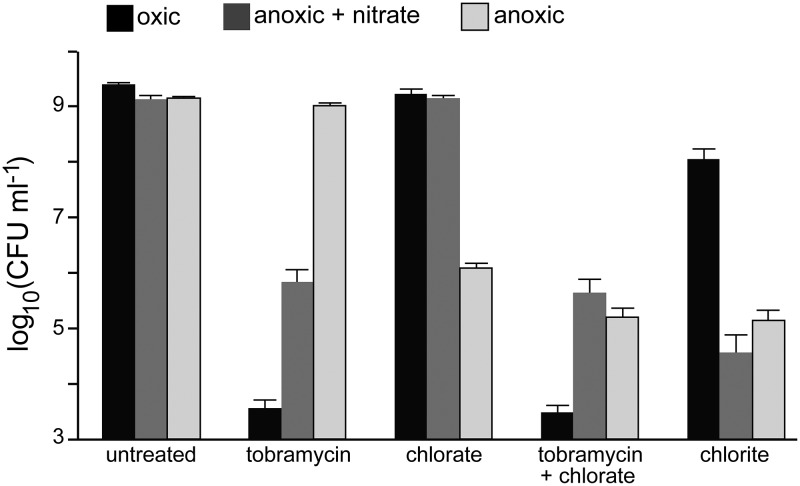
Chlorate kills oxidant-starved P. aeruginosa cells displaying physiological tolerance to tobramycin. Viable-cell plate counts from P. aeruginosa cultures that were incubated for 4 h without (untreated) or with 40 μg/ml tobramycin, 10 mM chlorate, 40 μg/ml tobramycin plus 10 mM chlorate, or 10 mM chlorite. Cultures were incubated with these compounds under oxic conditions (black), anoxic conditions with 40 mM nitrate (dark gray), or anoxic conditions without nitrate (light gray). Data show the means of results of 9 biological replicates from 3 independent experiments, and error bars indicate standard errors.

Because tobramycin is most effective at killing aerobically growing P. aeruginosa cells (42, 43), whereas chlorate is predicted to target cells containing Nar (25), we first determined whether these compounds were effective under different conditions of oxidant exposure. High-density P. aeruginosa cultures (∼10^9^ CFU ml^−1^) were incubated with and without drugs under oxic conditions, anoxic conditions with 40 mM nitrate, or anoxic conditions without nitrate (no terminal electron acceptor) for 4 h, after which viability was determined (Fig. 1). As predicted, oxic cultures are sensitive to tobramycin. Anoxic cultures are sensitive to tobramycin when supplied with nitrate but tolerant in the absence of an electron acceptor. Conversely, chlorate does not kill oxic cultures or anoxic cultures supplied with nitrate, while anoxic cultures lacking nitrate are sensitive to chlorate. These findings are consistent with those of prior studies demonstrating that cells require a minimum membrane potential for tobramycin uptake and efficacy (35, 36), which can be established by oxygen or nitrate respiration.

**FIG 2:**
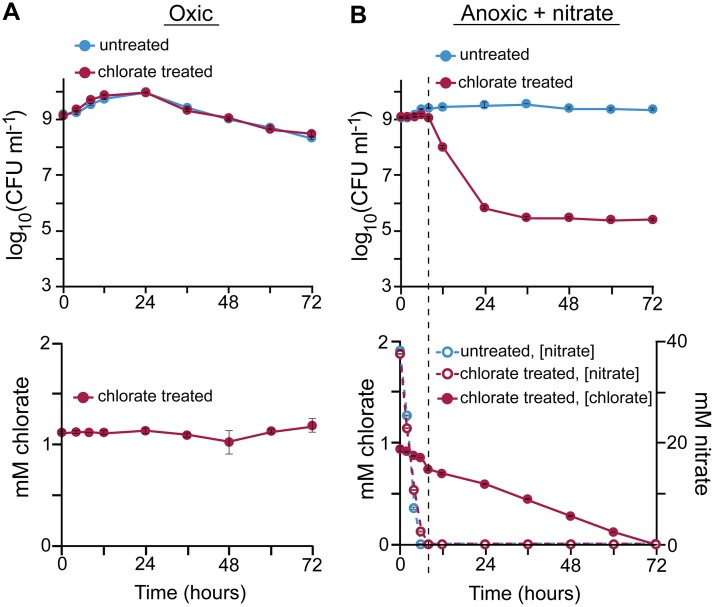
Chlorate consumption is correlated with cell death during oxidant starvation. P. aeruginosa cultures were incubated without (untreated) or with 1 mM chlorate (chlorate treated) under oxic conditions (A) or under anoxic conditions with 40 mM nitrate (B). Cultures were monitored for 72 h to determine viable-cell counts (top) and chlorate and nitrate concentrations (bottom) over time. The dashed line in panel B shows the time when nitrate concentrations approximate zero in chlorate-treated cultures. Data show the means of results from 3 biological replicates, and error bars indicate standard errors. In some cases, error bars are smaller than the size of the symbols.

Although anoxic cultures supplied with nitrate utilize Nar, these cells are chlorate tolerant (Fig. 1). However, nitrate concentrations in these experiments were 4-fold higher than chlorate concentrations. When nitrate is provided to anoxic cultures at low concentrations that approximate those found in CF patient sputum or chronic wounds (400 μM nitrate) (19, 23), tobramycin and chlorate lethality is indistinguishable from that in cultures lacking nitrate (see Fig. S1 in the supplemental material). We attribute this to the fact that under these conditions, 400 μM nitrate is completely consumed in a short time period (see Fig. 2B). Combined chlorate and tobramycin treatment under oxic conditions or anoxic conditions plus nitrate results in killing similar to that of tobramycin treatment alone, but combined treatment yields greater death under anoxic conditions (Fig. 1) (0.9 log_10_ CFU ml^−1^ more death with the combined treatment than with chlorate treatment; *t* test, *P < *0.0001). Lastly, we looked at sensitivity to chlorite, the predicted product of Nar-mediated chlorate reduction. Anoxic cultures are much more sensitive to chlorite than oxic cultures (Fig. 1). The mechanism of chlorite toxicity is not fully understood, although it and other reactive chlorine species are known to oxidize amino acids (62, 63), inducing cell death via protein aggregation (64). Cells with sufficient access to a respiratory oxidant may be better equipped to handle this stress because they have more energy for repair (65, 66).

10.1128/mBio.01400-18.1 FIG S1 Drug sensitivity with CF lung nitrate concentrations. Viable-cell plate counts from P. aeruginosa cultures incubated for 4 h without (untreated) or with 40 μg/ml tobramycin, 10 mM chlorate, 40 μg/ml tobramycin plus 10 mM chlorate, or 10 mM chlorite are shown. Cultures were incubated with these compounds under anoxic conditions with 400 μM nitrate or under anoxic conditions without nitrate. Note that data for anoxic conditions without nitrate are the same as those in Fig. 1 and included for ease of comparison. Data for anoxic conditions with 400 μM nitrate show the means from 3 biological replicates, and error bars indicate standard errors. Download FIG S1, PDF file, 0.35 MB. Copyright © 2018 Spero and Newman. 2018 Spero and Newman This content is distributed under the terms of the Creative Commons Attribution 4.0 International license.

### Chlorate toxicity requires chlorate consumption and is protected by respiration.

To determine whether chlorate reduction is linked to cell death, we monitored cell viability and chlorate concentrations in oxic cultures over 72 h. Under these conditions, chlorate concentrations are stable, and likewise, there is no chlorate-associated cell death (Fig. 2A). In anoxic cultures supplemented with 40 mM nitrate, nitrate is consumed quickly and undetectable within 8 h (Fig. 2B). During nitrate consumption, chlorate is consumed at a seemingly constant rate, although much more slowly than nitrate consumption. Chlorate-associated cell death does not occur until nitrate is consumed (dashed line in Fig. 2B). This result suggests that anaerobic respiration may protect cells from chlorate toxicity, perhaps by supplying energy required for chlorite detoxification or repair.

Changes in chlorate and nitrate concentrations can be attributed to cell activity because concentrations of these compounds are stable in abiotic controls (Fig. S2). Further, assuming a 1:1 stoichiometry of chlorate-chlorite converted by Nar, chlorite concentrations exceed our experimental detection limit (1 μM) at the observed rates of chlorate consumption. However, we did not detect chlorite in any of our experiments, potentially because chlorite reacts with intracellular components before measureable concentrations can accumulate. Similarly, other reactive chlorine species are known to react quickly with biomolecules (63).

10.1128/mBio.01400-18.2 FIG S2 Nitrate and chlorate concentrations are stable in cell-free growth medium. One millimolar chlorate concentrations were monitored in LB that was incubated for 72 h under oxic conditions (filled circles), anoxic conditions (open circles), and anoxic conditions with 40 mM nitrate (open circles). Forty millimolar nitrate concentrations were monitored in LB medium that was incubated for 72 h under anoxic conditions plus 1 mM chlorate (filled circles). Concentrations were monitored over time to show that they are stable in the absence of cells. Data show the means from three biological replicates, and error bars indicate standard errors. Download FIG S2, PDF file, 0.41 MB. Copyright © 2018 Spero and Newman. 2018 Spero and Newman This content is distributed under the terms of the Creative Commons Attribution 4.0 International license.

### Nar genes are required for chlorate consumption and toxicity.

**FIG 3:**
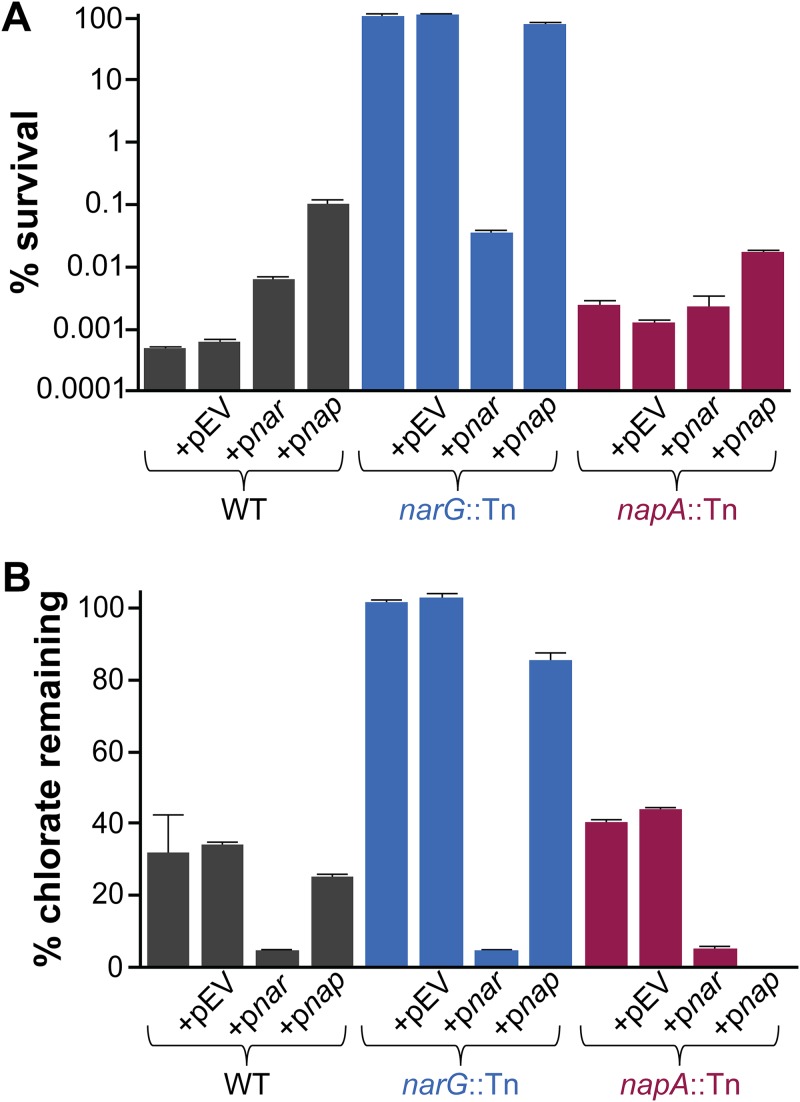
*nar* genes are required for chlorate reduction and sensitivity. Strains were grown aerobically, resuspended in fresh medium containing or lacking 1 mM chlorate, and incubated in an anaerobic glove box for 72 h. (A) The percent survival was calculated for each strain as the viable-cell counts in chlorate-treated cultures divided by the mean viable-cell count in untreated cultures at the end of the incubation, multiplied by 100. (B) The percentage of chlorate remaining was calculated for each strain as the concentration of chlorate in each culture at the end of the incubation divided by the initial concentration in the medium, multiplied by 100. Strains with pEV, p*nar*, and p*nap*, indicate strains that contain an empty vector, a vector with *narGHJI*, and a vector with *napEFDABC*, respectively. Three biological replicates of both treated and untreated cultures were used in this experiment, and error bars indicate standard errors.

Having found a correlation between chlorate consumption during oxidant starvation and cell death, we tested whether Nar is required for chlorate consumption and toxicity. In these experiments, we used the wild type (WT) and a *narG* transposon mutant and complemented these strains with *narGHJI* (encoding Nar structural subunits and assembly proteins) carried by an arabinose-inducible vector or with an empty vector. Because *narG* mutants cannot grow anaerobically, all strains were grown aerobically, washed and resuspended in fresh medium containing or lacking chlorate, and moved to an anaerobic chamber to adapt to anoxia. Viable-cell plate counts were determined after a 72-h incubation. While oxic WT cultures are not sensitive to chlorate (Fig. 1), anoxia-adapted cultures show a 5-log decrease in viable-cell counts compared to untreated cultures (Fig. 3A). The *narG* mutant, however, is resistant to chlorate; complementation with the *narGHJI* genes restores chlorate sensitivity to the *narG* mutant, while the empty vector has no effect. Likewise, chlorate concentrations are stable in *narG* mutant cultures over the course of the experiment, while chlorate concentrations decrease in WT and *narG* mutant-complemented cultures (Fig. 3B). This demonstrates that Nar is necessary and sufficient for chlorate reduction and its associated cell death.

Because P. aeruginosa can also reduce nitrate with the periplasmic nitrate reductase Nap (24), we tested whether Nap also contributes to chlorate sensitivity. *napA* insertion mutant strains consume 9% less chlorate than WT strains (with and without empty vector strains; *t* test, *P = *0.02) and have a 3-fold-higher percentage of survival (with and without empty vector strains; *t* test, *P  = *0.004). This suggests that periplasmic Nap may play a small role in chlorate reduction, which differs from findings of a prior report stating that this enzyme is incapable of chlorate reduction (53). When *nap* genes are overexpressed in a *narG* mutant background, 15% of the chlorate is consumed. Surprisingly, this is associated with very little cell death (78% survival). Indeed, all strains overexpressing *nap* genes consume more chlorate yet survive better than their empty-vector counterparts (*t* test, all *P < *0.02). Similarly, the WT strain overexpressing *nar* genes consumes more chlorate than its empty-vector counterpart (*t* test, *P < *0.0001), yet survives better (*t* test, *P = *0.002) (Fig. 3A). The relationship between chlorate reduction and death, thus, appears to be nuanced and may be influenced both by the cellular location of the chlorate reduction machinery and by the chlorate reduction rate.

### The Δ*lasR* strain has increased rates of nitrate respiration and increased chlorate sensitivity.

**FIG 4:**
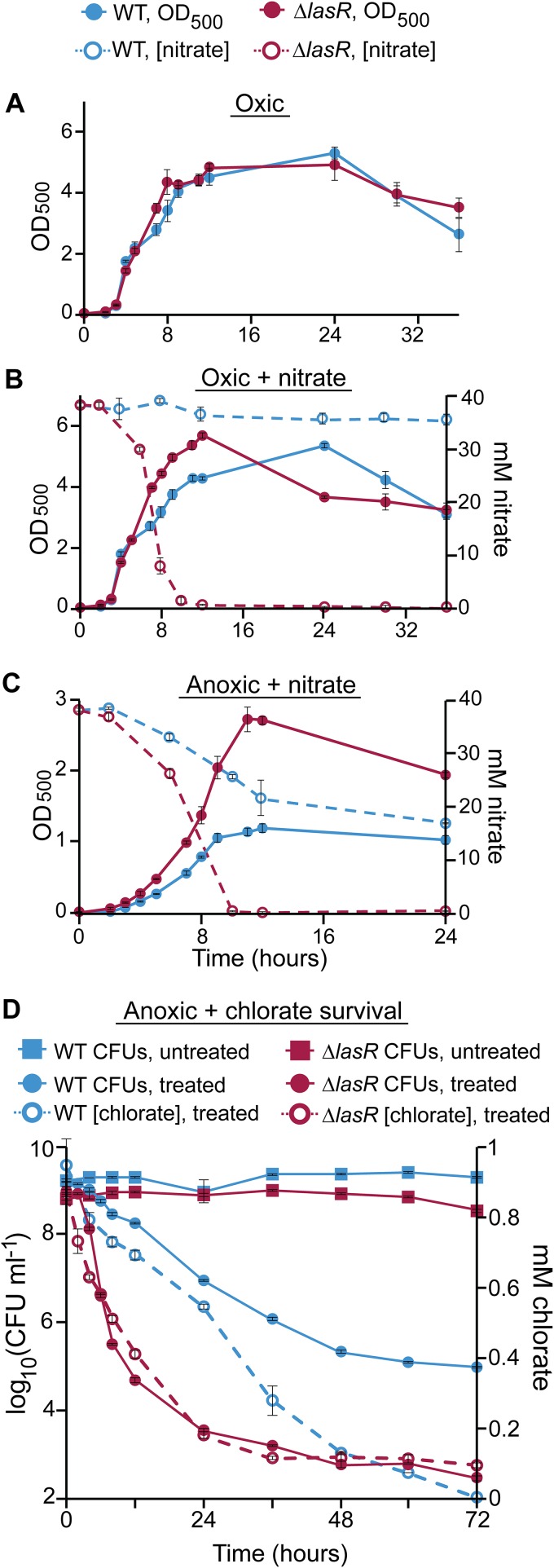
The Δ*lasR* mutant has increased rates of nitrate respiration and chlorate consumption and is more sensitive to chlorate. Cell density (OD_500_, filled circles) and nitrate concentrations (open circles) were monitored in the WT and Δ*lasR* mutant cultures growing under oxic conditions (A), oxic conditions with 40 mM nitrate (B), and anoxic conditions with 40 mM nitrate (C). (D) The WT and Δ*lasR* cultures were incubated without (squares) or with (filled circles) 1 mM chlorate under anoxic conditions for 72 h, over which time viable-cell counts (solid lines) and chlorate concentrations (dashed lines) were monitored. Data from all experiments show the means of results from three biological replicates, and error bars indicate standard errors.

Because P. aeruginosa *lasR* mutants have increased rates of Nar-mediated nitrate respiration under oxic and hypoxic conditions (51), we hypothesized that such mutants might be particularly chlorate sensitive. To test this, we compared the levels of growth and nitrate consumption of WT and Δ*lasR* strains under different conditions. The WT and the Δ*lasR* mutant grow similarly under oxic conditions (Fig. 4A), but upon addition of 40 mM nitrate, the Δ*lasR* mutant grows more quickly than the WT during late exponential/early stationary phase, though both cultures ultimately reach the same maximum cell density (Fig. 4B). This increased growth rate correlates with increased nitrate consumption by the *ΔlasR* mutant, which consumes all supplemented nitrate, whereas the WT consumes only 12% over 36 h (Fig. 4B). During anaerobic growth with 40 mM nitrate, the Δ*lasR* mutant also consumes nitrate more quickly than the WT and does so to completion, whereas the WT consumes only 50% over 24 h (Fig. 4C). Here, rapid and complete nitrate consumption allows anoxic Δ*lasR* cultures to grow faster and achieve higher cell densities than those of the WT. Finally, when incubated under anoxic conditions with chlorate, the Δ*lasR* mutant consumes chlorate more quickly than the WT, which correlates with increased rates of chlorate-associated cell death (Fig. 4D). Although similar amounts of chlorate were ultimately consumed by both strains, viable-cell counts were 100-fold lower in Δ*lasR* cultures at the end of the experiment, demonstrating that *lasR* mutants are particularly susceptible to anoxic chlorate treatment. In control experiments, we found that a Δ*lasR* Δ*narGHJI* strain is chlorate tolerant (Fig. S3) and shows WT levels of nitrate utilization (Fig. S4), demonstrating that chlorate sensitivity in the *lasR* strain is *nar* dependent.

10.1128/mBio.01400-18.3 FIG S3 *nar* genes are required for chlorate reduction and sensitivity in the Δ*lasR* mutant. Strains were grown aerobically, resuspended in fresh medium containing or lacking 1 mM chlorate, and incubated in an anaerobic glove box for 72 h. (A) The percent survival was calculated for each strain as the viable-cell counts in chlorate-treated cultures divided by the mean viable-cell count in untreated cultures at the end of the incubation, multiplied by 100. (B) The percent chlorate remaining was calculated for each strain as the concentration of chlorate in each culture at the end of the incubation divided by the initial concentration in the medium, multiplied by 100. Three biological replicates of both treated and untreated cultures were used in this experiment, and error bars indicate standard errors. Download FIG S3, PDF file, 0.58 MB. Copyright © 2018 Spero and Newman. 2018 Spero and Newman This content is distributed under the terms of the Creative Commons Attribution 4.0 International license.

10.1128/mBio.01400-18.4 FIG S4 Increased nitrate utilization in the Δ*lasR* mutant requires *nar* genes. Strains were grown aerobically in LB with 40 mM nitrate for 24 h, after which the percentage of nitrate remaining in the culture was determined (the final concentration divided by the initial nitrate concentration, multiplied by 100). Data show the means from three biological replicates, and error bars indicate standard errors. Download FIG S4, PDF file, 0.18 MB. Copyright © 2018 Spero and Newman. 2018 Spero and Newman This content is distributed under the terms of the Creative Commons Attribution 4.0 International license.

### Chlorate and tobramycin target distinct populations in aggregate biofilms.

**FIG 5:**
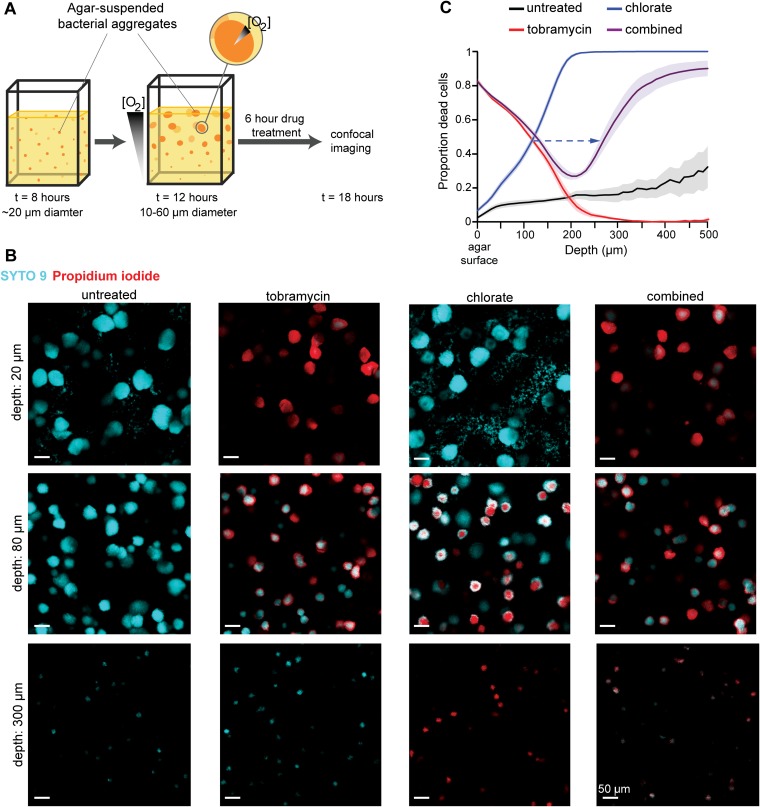
Tobramycin and chlorate target distinct populations in aggregate biofilms. (A) Cartoon of the agar block biofilm assay (ABBA), where cells suspended in agar medium grow as aggregate biofilms. At early incubations, aggregates are uniform in size, but oxygen gradients develop over time, both within the aggregate population and within individual aggregates, leading to a metabolically heterogeneous population. After 12 h of growth, aggregates are incubated with drugs for 6 h before they are stained and imaged via confocal microscopy. (B) Representative images of untreated aggregates and those treated with 40 μg/ml tobramycin, 10 mM chlorate, or 40 μg/ml tobramycin plus 10 mM chlorate (combined) are shown at three depths, where cells are stained with SYTO 9 (cyan; live) and propidium iodide (red; dead). The scale bar is 50 μm for all images. (C) Confocal images were used to generate a sensitivity profile for each treatment condition, where the proportion of dead cells (propidium iodide intensity divided by the sum of the propidium iodide and SYTO 9 intensities) was determined at each depth. The dashed arrow highlights a shift in the depth, where 50% of cells are killed by chlorate in chlorate-only samples and compared to combined-treatment samples. Data show the means from 6 independent experiments, and error bars indicate standard errors.

To assess whether our findings in planktonic cultures might apply to a biofilm mode of growth that approximates that found *in vivo* (40), we used an agar block biofilm assay (ABBA) to study aggregate biofilms. In the ABBA system, an overnight culture is diluted into 0.5% molten agar medium, allowed to solidify, and incubated overnight at 37°C. Agar-suspended cells grow as aggregates, which develop into metabolically distinct populations at different spatial scales. Over time, aggregates near the top of the agar grow more quickly than aggregates deeper in the agar because they consume oxygen that is enriched at the surface, which decreases oxygen availability to deeper aggregates (67, 68) (Fig. 5A). Oxygen gradients can also develop within large aggregates, where cells on the exterior scavenge oxygen before those on the interior can access it (69). We predicted that biofilm cells with access to oxygen are tobramycin sensitive and chlorate tolerant but that cells that are oxygen starved are tobramycin tolerant and chlorate sensitive.

P. aeruginosa aggregates were grown overnight in Luria-Bertani medium (LB) agar supplemented with 5 mM nitrate, after which they were treated for 6 h by pipetting LB with or without tobramycin, chlorate, or both compounds on top of the agar blocks. Following treatment, aggregates were stained with SYTO 9 and propidium iodide (PI) and imaged via confocal microscopy (Fig. 5B and Movie S1 to S4). SYTO 9 is membrane permeable and stains all cells, whereas PI is membrane impermeable and, thus, thought to enter nonviable cells with damaged membranes where it displaces SYTO 9 (70). Though PI can stain viable cells that grow slowly and have a weak membrane potential (71), because a relatively small proportion of cells stain with PI in untreated samples (Fig. 5C), we interpret PI- or SYTO 9-stained cells as dead or alive in response to drug treatment, respectively. We found that cells in surface aggregates are killed by tobramycin, whereas those at depth are tobramycin tolerant (Fig. 5B). The pattern corresponds to the expected profile of oxygen availability (67). Cells in middepth aggregates display graded sensitivity at the individual aggregate scale, with cells on the exterior being killed and cells on the interior staying alive. Chlorate targets the opposite population: surface aggregates and the exterior of middepth aggregates are chlorate tolerant, whereas aggregates at depth and the interior of middepth aggregates are killed (Fig. 5B). These findings are consistent with those of our planktonic studies (Fig. 1), where cells with or without access to oxygen were found to be killed by tobramycin or chlorate, respectively.

10.1128/mBio.01400-18.5 MOVIE S1 Movie through a confocal z-stack of an aggregate biofilm population with increasing depth. Cyan staining (SYTO 9) indicates live cells, and red staining (propidium iodide) indicates dead cells. Download Movie S1, MOV file, 1.5 MB. Copyright © 2018 Spero and Newman. 2018 Spero and Newman This content is distributed under the terms of the Creative Commons Attribution 4.0 International license.

10.1128/mBio.01400-18.6 MOVIE S2 Movie through a confocal z-stack of a tobramycin-treated aggregate biofilm population with increasing depth. Cyan staining (SYTO 9) indicates live cells, and red staining (propidium iodide) indicates dead cells. Download Movie S2, MOV file, 1.3 MB. Copyright © 2018 Spero and Newman. 2018 Spero and Newman This content is distributed under the terms of the Creative Commons Attribution 4.0 International license.

10.1128/mBio.01400-18.7 MOVIE S3 Movie through a confocal z-stack of a chlorate-treated aggregate biofilm population with increasing depth. Cyan staining (SYTO 9) indicates live cells, and red staining (propidium iodide) indicates dead cells. Download Movie S3, MOV file, 1.6 MB. Copyright © 2018 Spero and Newman. 2018 Spero and Newman This content is distributed under the terms of the Creative Commons Attribution 4.0 International license.

10.1128/mBio.01400-18.8 MOVIE S4 Movie through a confocal z-stack of a tobramycin- and chlorate-treated aggregate biofilm population with increasing depth. Cyan staining (SYTO 9) indicates live cells, and red staining (propidium iodide) indicates dead cells. Download Movie S4, MOV file, 1.3 MB. Copyright © 2018 Spero and Newman. 2018 Spero and Newman This content is distributed under the terms of the Creative Commons Attribution 4.0 International license.

### Drug treatment alters oxygen gradients and drug sensitivity.

Given that tobramycin and chlorate can target and kill distinct, complementary populations within metabolically stratified biofilms, we anticipated that combined tobramycin and chlorate treatment might compromise all cells within aggregate populations, but paradoxically, this proved not to be the case (Fig. 5B). In combined-treatment samples, aggregates at the surface were killed by tobramycin and aggregates at depth were killed by chlorate, but middepth aggregates stained similarly to tobramycin-only-treated samples (exterior killed, interior alive). Quantifying these ABBA live/dead profiles, we find that the combined treatment profile aligns with the tobramycin-only profile for the first 180 μm but that the depths where chlorate kills shifts: the depth at which 50% of the population is killed by chlorate is 162 ± 14 μm (mean ± standard error of the mean [SEM]) deeper in combined-treatment samples than in chlorate-only-treated samples (dashed arrow in Fig. 5C). We speculated that this shift might reflect changes in oxygen availability due to changes in cellular consumption rates, which might explain why combined treatment did not eradicate all cells.

**FIG 6:**
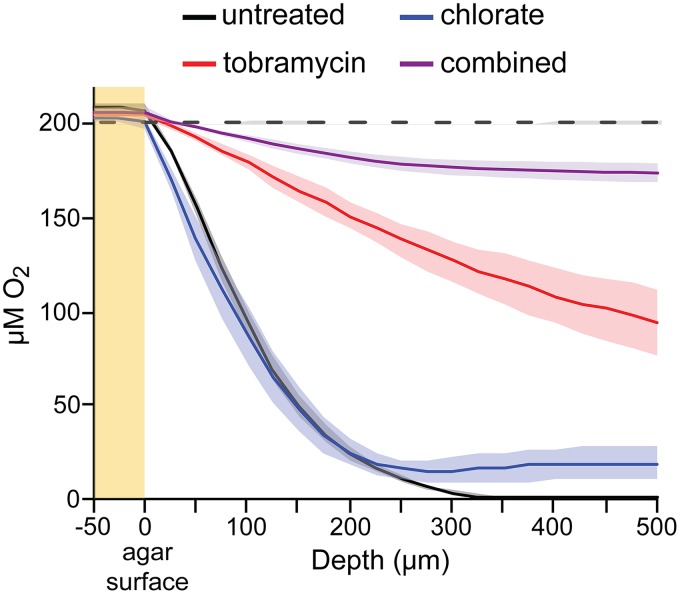
Oxygen profiles in treated aggregate biofilm populations. Oxygen profiles of P. aeruginosa aggregate biofilms after a 6-h incubation without a treatment (black) or with 40 μg/ml tobramycin (red), 10 mM chlorate (blue), or 40 μg/ml tobramycin plus 10 mM chlorate (combined; purple). The yellow region highlights measurements taken above the surface of the agar, and the dashed black line shows oxygen profiles from uninoculated agar samples. Data show the means from 3 independent experiments, and colored regions indicate standard errors.

To test this hypothesis, we measured oxygen profiles in ABBA samples (Fig. 6). In untreated samples, oxygen concentrations decrease until anoxia occurs at a depth of about 350 μm. However, tobramycin-treated samples never become anoxic; oxygen concentrations remain at around 50% of atmospheric values at even the greatest depths. This observation supports our hypothesis that the tobramycin-mediated death of surface and exterior cells allows for increased oxygen penetration, leading some populations in combined-treatment samples to shift from a chlorate-sensitive to a -tolerant state (depths of 120 to 220 μm) (Fig. 5C). Increased oxygen penetration also correlates with increased survival in tobramycin-only-treated samples compared to that in untreated samples at depths of >200 μm (Fig. 5C). Oxygen consumption rates for equivalent SYTO-9-stained cells are lower in tobramycin-only-treated (0.27 μM/μm) than in untreated (0.50 μM/μm) samples at depths of 150 to 200 μm (Fig. 6), which may explain why tobramycin-treated cells remain viable at these depths. In chlorate-treated samples, oxygen concentrations decrease until about 200 μm but remain greater than zero (Fig. 6). This is consistent with our finding that most cells at depths of >200 μm are dead in chlorate-treated samples (Fig. 5C). As was expected, the combined-treatment samples are relatively oxygen replete (Fig. 6), presumably because few cells survive to respire oxygen.

Though we expected deep aggregates in tobramycin-treated samples to die over prolonged exposure or that sequential treatment with chlorate and then tobramycin (or vice versa) would kill all cells given enough time, we were unable to perform these experiments because the ABBA system is not amenable to incubations greater than a day (i.e., a large proportion of cells in the untreated controls stain with PI after ∼20 h of incubation). Such experiments require a different experimental setup and are priorities for future work.

## DISCUSSION

Nar-mediated nitrate respiration is commonly used by bacterial pathogens to adapt to hypoxic/anoxic host environments, marking Nar as a potential therapeutic physiological target for treating oxidant-limited infections. While it has long been known that Nar-mediated chlorate reduction is toxic to bacteria (72, 73), this is the first study to explore chlorate as a prodrug against antibiotic-tolerant pathogens. We show that Nar-mediated chlorate reduction in the absence of an electron acceptor kills P. aeruginosa cells that tolerate the aminoglycoside tobramycin. Within metabolically stratified aggregate biofilms, chlorate and tobramycin kill distinct populations, targeting cells in the hypoxic/anoxic and oxic zones, respectively. Tobramycin treatment perturbs the biofilm microenvironment by increasing oxygen penetration, which increases survival and removes chlorate sensitivity for certain members of the population. These findings demonstrate chlorate’s potential therapeutic value and provide insight into how the physiological adaptation of pathogens plays a central role in drug efficacy.

We used P. aeruginosa in this study as a representative facultative anaerobe to begin to explore chlorate’s potential as a prodrug. We were able to leverage knowledge from prior work regarding how P. aeruginosa adapts to host environments to inform our experimental design. Given that *lasR* mutants are frequently isolated from P. aeruginosa-infected patients (48–50), they are more resistant to antibiotics commonly used to treat such infections (51, 52), and they have a propensity for Nar-mediated nitrate respiration (51), we explored the use of chlorate for killing this recalcitrant genetic population (46, 49, 51). Our finding that chlorate is more effective at killing the *lasR* mutant than the WT suggests that it may be particularly well suited to combat this clinically relevant mutant. The presence of *lasR* mutants in chronic wounds, blood infections, and the lungs of CF and pneumonia patients (46, 48, 50) suggests that this adaptation benefits P. aeruginosa in many host environments. However, because the primary mechanism of chlorate resistance in P. aeruginosa is a Nar-inactivating mutation (74), adaptation to the drug might severely compromise pathogen fitness *in vivo* (3).

Antibiotic treatment failure is often not attributable to genetic resistance, since patient isolates often show *in vitro* sensitivity to the administered antibiotic (75). Rather, oxidant-starved pathogen populations, such as those found in biofilms, may play a critical role in antibiotic tolerance and treatment failure (43). A number of strategies, including treatments that disrupt the proton motive force or cell membrane (76), reactivate cell metabolism (77), or activate a destructive pathway (78), are being explored for targeting this metabolic state. Colistin in known to kill oxidant-starved P. aeruginosa cells (41); however, while inhaled colistin therapy is widely used to treat chronic CF patient lung infections, whether its chronic use benefits patients is unclear (79). Azithromycin is also known to kill oxidant-starved P. aeruginosa cells (80), and while its use is associated with positive outcomes for CF patients, its chronic use has been associated with multidrug-resistant nontuberculosis mycobacterial infections (79, 81). In the case of CF, chronic tobramycin treatment remains the standard of care for patients with persistent P. aeruginosa infections (79), despite its inability to eradicate anaerobic populations. Thus, the potential for chlorate to kill oxidant-limited pathogens that tolerate tobramycin is worth exploring.

Given that many facultative anaerobic pathogens carry *nar* (1) and anoxia alone stimulates its expression in many of these organisms (25, 82, 83), chlorate might have broad potential application. One attractive aspect of chlorate is that, unlike antibiotics that are designed to inhibit an essential process (84), chlorate coopts the activity of its target to produce a toxic by-product. Thus, even though pathogens may employ other energetic schemes during anaerobic growth and survival (85, 86), chlorate-susceptible pathogens need only synthesize Nar rather than rely on it for survival. Such “Trojan horse” strategies have been explored in other contexts for bacteria and other pathogens (87, 88). Because Nar catalyzes the majority of chlorate reduction and is oriented toward the cytoplasm, we might anticipate that chlorate reduction does not harm neighboring cells lacking Nar (e.g., mammalian cells). Consistent with this prediction, chlorite did not accumulate in our planktonic cultures, nor did we find evidence that chlorate reduction in the anoxic zone of our biofilm populations harmed those in the oxic zone. However, it is possible that a toxic by-product other than chlorite is produced or released by chlorate-reducing cells that we did not detect. Going forward, it will be important to determine the mechanism of chlorate toxicity and which chlorine reduction products are involved, the extent to which chlorate-mediated killing is specific to target cells, the effective *in vivo* chlorate concentrations, and whether our *in vitro* results translate into positive outcomes in *in vivo* models.

Lastly, our results show that drug treatment can perturb the microenvironment around pathogens in such a way as to modulate their susceptibility to other drugs. Oxygen availability drives P. aeruginosa sensitivity to chlorate, tobramycin, and other antibiotics (32), so finding that tobramycin treatment itself increases oxygen availability to other members of the biofilm population is striking. When tobramycin treatment kills aerobically respiring populations on the biofilm exterior, it benefits cells in hitherto-hypoxic regions by increasing their access to oxygen, which in turn renders them, at least temporarily, tolerant to both tobramycin and chlorate. Ultimately, a direct examination of the energetic states that exist within a biofilm population, how they change over time, and how they respond to drug perturbations will be necessary to understand and effectively respond to physiological antibiotic tolerance.

## MATERIALS AND METHODS

### Bacterial strains and growth conditions.

Strains used in this study include P. aeruginosa PA14 (wild type [WT]), isogenic *narG* and *napA* transposon insertion mutants (25), and an isogenic, in-frame, markerless *lasR* deletion mutant (65). Strains that were constructed for this work are described below. All experiments used Luria-Bertani medium (LB; Difco) as a growth medium, supplemented with KNO_3_ as specified. Aerobic liquid cultures were incubated at 37°C with shaking at 250 rpm. Anaerobic work was conducted in an anaerobic glove box with a 95% N_2_–5% H_2_ atmosphere, and anaerobic cultures were incubated at 33°C.

### Bacterial-survival assays and viability measurements.

In aerobic-survival endpoint assays, overnight aerobic cultures were pelleted, washed twice with and resuspended in LB, and added to a 96-well microtiter plate at high cell density (optical density at 500 nm [OD_500_] ∼ 3). Wells also contained sodium chlorate (final concentration, 10 mM), sodium chlorite (final concentration, 10 mM), tobramycin (final concentration, 40 μg ml^−1^), or an equal volume (20 μl) of dH_2_O in untreated-control wells (200-μl total volume per well). Ninety-six-well plates were incubated at 37°C with shaking at 250 rpm for 4 h before viable cells on plates were counted. Anaerobic-survival endpoint assays were performed as described above, except that overnight cultures were grown anaerobically at 33°C in LB with 40 mM KNO_3_, cells were washed and resuspended in either LB or LB with 40 mM KNO_3_ under anaerobic conditions, and 96-well plates were incubated anaerobically at 33°C for 4 h and then removed from the glove box so that cultures could be serially diluted for viable-cell plate counts.

Aerobic- and anaerobic-survival assays were also conducted over the course of 72 h. The experimental setup was the same as described above, except that culture volumes were 5 ml (in capped tubes), cultures were treated with 1 mM sodium chlorate, and anaerobic experiments using LB with 40 mM KNO_3_ were buffered with 200 mM MOPS (morpholinepropanesulfonic acid) to prevent an increase in culture pH that results from denitrification reactions. Two-hundred-microliter samples were taken over time and used for viable-cell plate counts and to quantify nitrate and chlorate concentrations (described below).

Viable-cell plate counts were performed by serially diluting samples in phosphate-buffered saline (PBS). Dilutions spanning 7 orders of magnitude were plated on LB agar plates as 10-μl drips. Plates were incubated at 37°C for ∼20 h, and incubation continued at room temperature (72 h of total incubation) to allow for the growth of slow-growing colonies. Colonies were counted daily, and numbers of CFU per milliliter were calculated at the end of the incubation period. All viable-cell plate counting was carried out under aerobic conditions.

### Nitrate and chlorate quantification.

Culture samples were centrifuged at 16,000 × *g* at room temperature for 10 min to pellet cells. Supernatants were diluted 1:10 in dH_2_O and added to 0.5-ml vials (Thermo Fisher Scientific catalog numbers 038010 and 038011). Nitrate and chlorate concentrations were quantified using the Dionex ICS 2000 ion chromatography system (Environmental Analysis Center, Caltech). Samples were loaded via a 15-μl sample loop onto an AS-19 separator (2- by 250-mm) column protected by an AG-19 guard (2 by 50 mm), maintained at 30°C. A hydroxide gradient was produced using a potassium hydroxide eluent generator cartridge and pumped at 0.25 ml per minute. The gradient began with a 10 mM hold for 10 min, increased linearly to 58 mM at 25 min, and remained at 58 mM until the end of data acquisition at 32 min. Seven minutes was allowed between analyses to return the column to initial conditions. Anions were detected at neutral pH using an AERS 500 2-mm suppressor (Thermo Fisher Scientific) operated in eluent recycle mode with an applied current of 30 mA, and the conductivity detection cell was maintained at 35°C. A carbonate removal device (CRD 200, 2 mm) was installed between the suppressor eluent out port and the conductivity detector eluent in port. Sodium chlorate and potassium nitrate standards were used to identify sample analytes via retention time and to generate standard curves for quantifying analyte concentrations. Similar methods were used to quantify chlorite concentrations, but chlorite was not detected in culture samples (1 μM detection limit).

### Construction of *nar* and *nap* complement strains and *narGHJI* mutant strains.

To construct complement strains, the *narGHJI* genes were amplified from P. aeruginosa using primers 5′-CCATACCCGTTTTTTTGGGCTAGCGAATTCGAGCTCAGGAGGAGATCAAGATGAGTCACC-3′ and 5′-GCAAATTCTGTTTTATCAGACCGCTTCTGCGTTCTGATTTAAGGTTCAGGCAGGACGTTT-3′, and the *napEFDABC* genes were amplified using primers 5′- CCATACCCGTTTTTTTGGGCTAGCGAATTCGAGCTCAGGAGGCTGGGCCAATGAACGAAC-3′ and 5′- GCAAATTCTGTTTTATCAGACCGCTTCTGCGTTCTGATTTAAGCGCTACCAGCCCTTCAC-3′. The fragments were each cloned into HindIII-digested pMQ72 (89) downstream of the arabinose-inducible promoter P*_ara_* via Gibson cloning (NEB number E2611) (90). The resulting plasmids, pMQ72-*narGHJI* and pMQ72-*napEFDABC*, and the empty vector were transformed into Escherichia coli DH10B cells. These plasmids and the empty vector were then introduced into P. aeruginosa WT and mutant strains via triparental conjugation, and successful exconjugants were selected by plating cells on VBMM medium (91) supplemented with 50 μg ml^−1^ gentamicin.

The *narGHJI* genes were deleted in WT PA14 and the isogenic Δ*lasR* background. The region upstream of *narG* was amplified using primers 5′-TAAAACGACGGCCAGTGCCACGTACTGGGTGTTCGCCCTG-3′ and 5′-CGCGCAGGGTCTTGATCTCCTCACCCGGTC-3′, and the region downstream of *narI* was amplified using primers 5′-GGAGATCAAGACCCTGCGCGGCCGGCGCAT-3′ and 5′-CATGATTACGAATTCGAGCTGCTGGCGCGGCAGGAAGCGC-3′. These fragments were cloned into HindIII- and SacI-digested pMQ30 (89) via Gibson cloning. The resulting plasmid was transformed into E. coli DH10B and introduced into P. aeruginosa strains via triparental conjugation, and merodiploids were selected by plating them on VBMM medium (91) supplemented with 50 μg ml^−1^ gentamicin. Δ*narGHJI* mutants were generated by resuspending merodiploid cells in PBS and plating them on LB supplemented with 10% sucrose. Correct mutants were confirmed both via PCR and their inability to grow anaerobically with nitrate.

### Anaerobic adaptation and survival.

Overnight cultures were grown aerobically in LB with 40 mM KNO_3_, 100 mM l-arabinose, 50 μg ml^−1^ gentamicin (for strains containing pMQ72-derived plasmids), and 50 μg ml^−1^ kanamycin (for transposon insertion strains). Overnight cultures were pelleted, washed twice in LB with 100 mM l-arabinose to remove antibiotics, and resuspended in LB with 100 mM l-arabinose with or without 1 mM sodium chlorate at high cell density (OD_500_ ∼ 3). Two hundred microliters of resuspended culture was added to 96-well microtiter plates, and the plates were moved to the anaerobic glove box for cells to adapt to the anaerobic lifestyle. After a 72-h incubation at 33°C, 96-well plates were removed from the anaerobic glove box and viable cells on plates were counted to calculate numbers of CFU per milliliter (described above). Percent survival was calculated for each strain by dividing CFU-per-milliliter values from cultures containing chlorate by the average CFU-per-milliliter value from control cultures lacking chlorate and multiplying by 100. CFU-per-milliliter values of untreated control cultures were similar across strains, ranging from 1.0 × 10^9^ to 2.2 × 10^9^. The percentage of chlorate remaining after the 72-h incubation was determined for each strain by dividing the final chlorate concentration in each culture by the initial chlorate concentration in the medium and multiplying by 100.

### ABBA.

Overnight aerobic cultures were diluted to an OD_500_ of 0.001 in molten agar growth medium that had been cooled to 44°C. For all agar block biofilm assays (ABBAs), the growth medium used was LB with 5 mM KNO_3_ and 0.5% noble agar. A portion of the dilution (175 μl) was added to chambered cover glass slides (Thermo Fisher Scientific number 155409), solidified at room temperature, and incubated at 37°C for 12 h in a humidified chamber. After incubation, cells that were not suspended in the agar were removed by adding 400 μl PBS to the top of each agar block and gently pipetting. Liquid was removed by inversion, and the wash was repeated. Aggregate cells were then treated by pipetting 125 μl of 24 mM sodium chlorate (final concentration, 10 mM), 125 μl 96 μg ml^−1^ tobramycin (final concentration, 40 μg ml^−1^), or 62.5 μl of 48 mM chlorate and 62.5 μl of 192 μg ml^−1^ tobramycin in combination (10 mM and 40-μg-ml^−1^ final concentrations, respectively) on the top of the agar block. All chlorate and tobramycin solutions were made in LB, and 125 μl LB was pipetted on the top of untreated control agar blocks. Treated and untreated samples were incubated at 37°C in a humidified chamber, with shaking at 220 rpm for 6 h. After incubation, samples were inverted to remove liquid, and cells were stained using a *Bac*Light LIVE/DEAD bacterial viability staining kit (Thermo Fisher Scientific number L7012). One hundred twenty-five microliters of staining solution (1 μl 3.34 mM SYTO 9, 1 μl 20 mM propidium iodide, 123 μl dH_2_O) was added to each sample, and samples were incubated at room temperature on a VWR variable-speed rocker at the highest speed for 90 min before being imaged.

### Confocal microscopy, image analysis, and staining quantification.

Before being imaged, ABBA samples were inverted to remove the staining solution, and 75 μl of a 1:50 dilution of 5-μm fluorescent beads (Spherotech; CFP-5045-2) was added to each sample. Excess liquid was removed by wicking, and beads were used to mark the surface of each sample. ABBA aggregates were imaged using a Leica TCS SPE confocal microscope with an ACS APO 0.3-numeric-aperture/10× objective. The agar surface was determined by visualizing fluorescent beads using a 405-nm solid-state laser for excitation, with data collected from 425 to 475 nm. LIVE/DEAD data were collected using a 488-nm solid-state laser for excitation, with emission collected at 510 to 550 nm and 610 to 650 nm for SYTO 9 and propidium iodide, respectively. Images were collected from three distinct locations near the center of each well as 500-µm z-stacks (50 slices total, 10-µm step size). z-stacks were collected in 8-bit mode with a scan format of 512 by 512 pixels (for quantification) or 1,024 by 1,024 pixels (for representative images) and line averaging of 2. Representative images were chosen from 6 independent experiments.

To quantify ABBA staining, images were loaded in Fiji (92), and background was subtracted from each image using the rolling ball method with a radius of 100 pixels. A threshold was applied to all images to exclude pixels with a value of <20, and total pixel intensity for both stains was determined for each image using the RawIntDen value in the Integrated Density measurement function. The proportion of sensitive cells was determined for each image (at each depth) by dividing the propidium iodide integrated density by the sum of the propidium iodide- and SYTO 9-integrated densities. ABBA staining quantifications are averages from 6 independent experiments (3 technical replicates per independent experiment).

### Oxygen profiling.

Oxygen profiles were determined from treated ABBA samples (12-h growth and 6-h treatment incubations; see above). ABBA samples were kept in a 37°C water bath during oxygen profiling experiments. Oxygen profiles were measured using a Clark-type amperometric electrode with a 25-μm-tip diameter that was connected to a picoampere amplifier in a multimeter (Unisense, Denmark), as described previously (21). Briefly, the microsensor was calibrated using a 37°C oxygen-free solution (0.1 M sodium hydroxide, 0.1 M sodium ascorbate) to obtain a zero-point reading and a 37°C air-saturated, 1% salt solution corresponding to 199.4 μM oxygen. The microsensor was manipulated via a motorized micromanipulator and was positioned 125 to 150 μm above the air-agar interface prior to data collection. The air-agar interface (depth = 0) was defined as the depth at which microsensor values decreased by >0.5% from its baseline value and was determined by moving the microsensor in 10-μm incremental steps. Profiling data were acquired using SensorTrace Pro 3.1.3 software, with which oxygen was measured at intervals of 25 μm for a total depth of 700 μm. Measuring time at each depth was set at 3 s, with 2 s between measuring points. Oxygen profile data are averages from 3 independent experiments (3 technical replicates per independent experiment).
