# Supplementary figures and images for: Chlorate Specifically Targets Oxidant-Starved, Antibiotic-Tolerant Populations of Pseudomonas aeruginosa Biofilms
Source: mBio. 2018 Sep 25;9(5):e01400-18. doi: 10.1128/mBio.01400-18 (PMC6156191; doi:10.1128/mBio.01400-18)

anoxic + 400  $\mu$ M nitrate

anoxic no nitrate

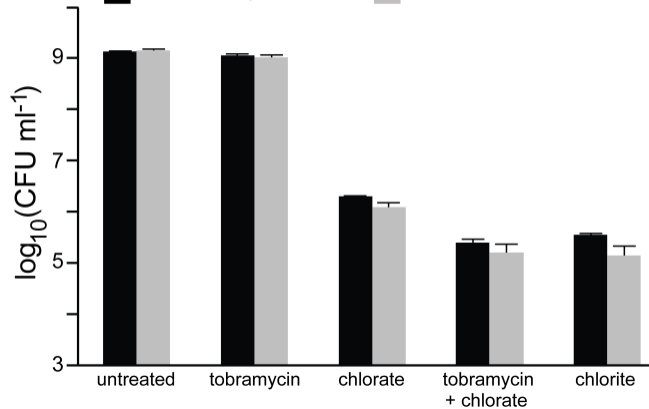

Supplement: FIG S1 [file mbo005184084sf1.pdf]

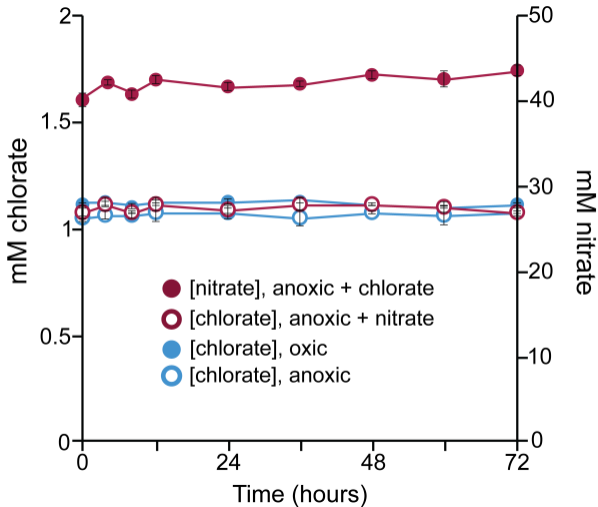

Supplement: FIG S2 [file mbo005184084sf2.pdf]

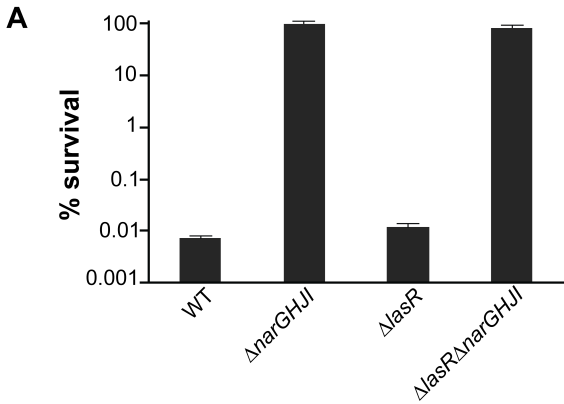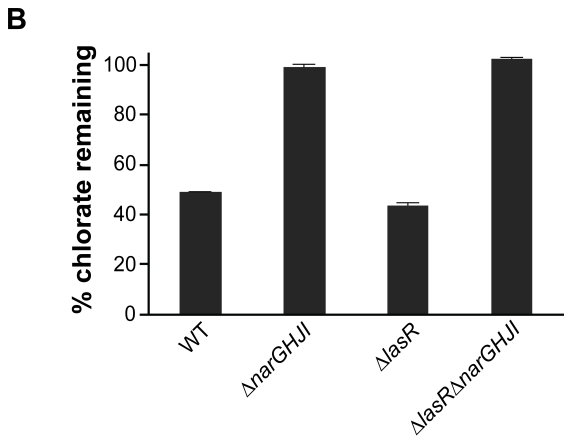

Supplement: FIG S3 [file mbo005184084sf3.pdf]

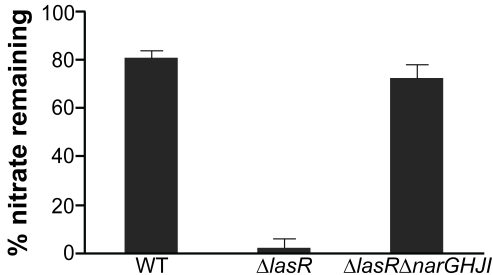

Supplement: FIG S4 [file mbo005184084sf4.pdf]
